# The Reliability, Validity and Applicability of Two Sport-Specific Power Tests in Synchronized Swimming

**DOI:** 10.2478/v10078-012-0030-8

**Published:** 2012-05-30

**Authors:** Mia Peric, Natasa Zenic, Gordana Furjan Mandic, Damir Sekulic, Dorica Sajber

**Affiliations:** 1 University of Split, Faculty of Kinesiology, Split, Croatia.; 2 University of Zagreb, Faculty of Kinesiology, Zagreb, Croatia.; 3 University of Ljubljana, Faculty of Sport, , Ljubljana, Slovenia.

**Keywords:** test construction, field testing, factor analysis, synchro

## Abstract

Sport-specific tests are rarely investigated in synchronized swimming (synchro). The aim of this research was to study the reliability and the validity of two sport-specific tests that are based on synchro elements, namely, the Barracuda thrust (“Barracuda”) and the Boost. The Barracuda is a move in which the swimmer begins in the back pike position (head down with the legs perpendicular to the surface of the water) and then moves the legs and hips rapidly upward, unrolling the body to obtain a maximal vertical position above the surface of the water. The Boost occurs when the swimmer rises rapidly out of the water, head first, to bring as much of the body as possible above the surface of the water. Both patterns are considered power moves and are therefore theoretically related to explosive strength. This study involved 22 female competitive synchro swimmers aged 16–18 years. The variables examined included performance on the Barracuda, Boost and countermovement jump and anthropometric measures (body height, body weight and body composition). Statistical analyses showed appropriate reliability for all tests, with no systematic bias between trials. A factor analysis calculated for the Barracuda, Boost and countermovement jump revealed one significant factor based on the Guttmann-Kaiser criterion with all three tests significantly projected. The structure of the significant factor did not change if the results for the Boost and Barracuda were normalized for body height. The Boost and Barracuda, but not the countermovement jump, were significantly correlated with the competitive achievements of the swimmers. In conclusion, the Boost and Barracuda are reliable and valid measures of the explosive strength of synchronized swimmers and are significantly related to competitive achievement.

## Introduction

**Figure 1:**
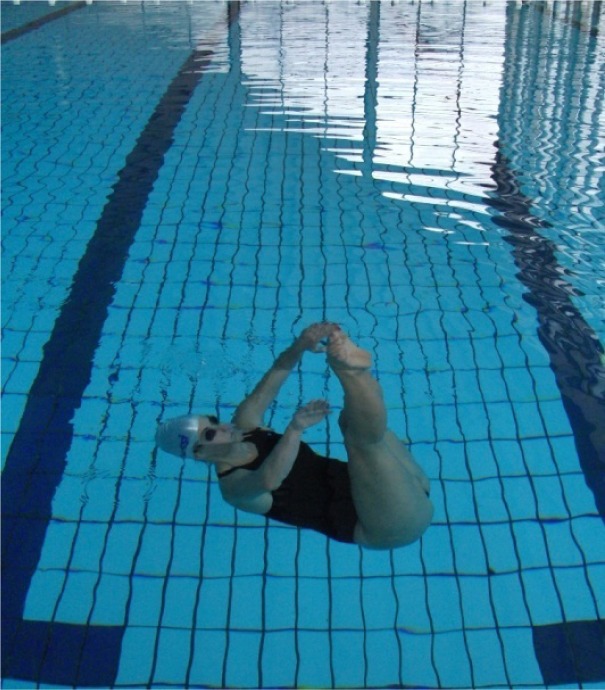

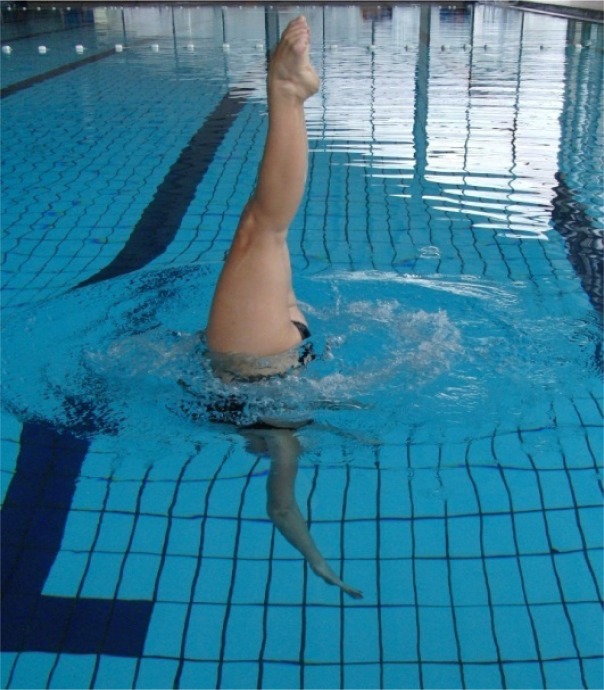
Barracuda performance Figure 1a. Start position Figure 1b. Finish position

**Figure 2:**
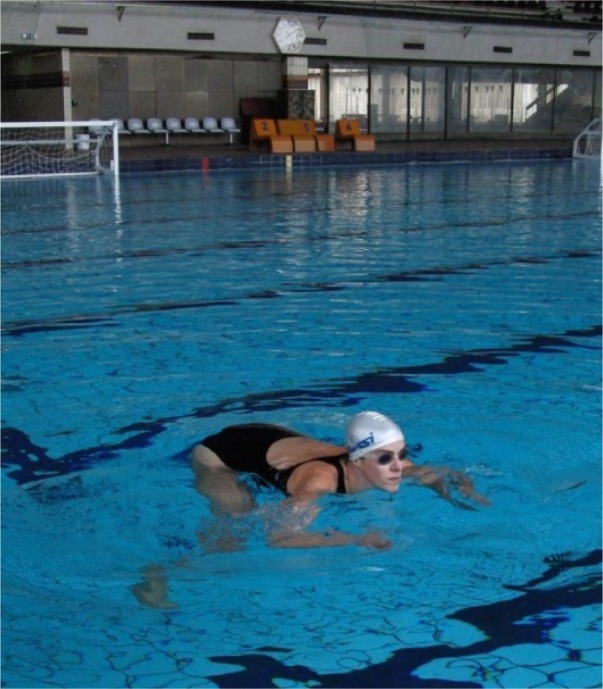

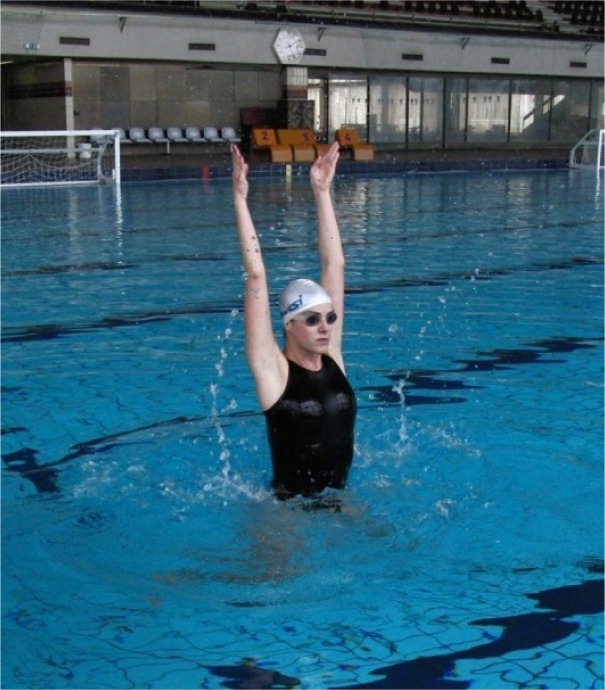
Boost performance Figure 2a. Start position Figure 2b. Finish position

Synchronized swimming (synchro) is an Olympic sport that is a hybrid form of swimming, dance, and ballet. Swimmers (in solos, duets, or teams) perform a synchronized routine of elaborate moves in and under the water accompanied by music (Zenic et al., 2010). Synchro demands advanced skills and requires great strength, endurance, flexibility, grace, artistry, and precise timing (Bante et al., 2007; Gabrilo et al., 2011). In a recent study, Bante et al. (2007) defined the VO_2_ and blood lactate values for comens (age 13.8 years) and seniors (22.6 years) after a simulated synchronized swimming routine. In short, the data showed 37.4±2.7 vs. 40.5±2 mm/kg/min (of VO_2_); 81.8±3.1% vs. 85.8±2.7% (of VO_2_peak); 5.7±0.9 vs. 4.5±0.4 mmol/l (of blood lactate, for comens and seniors, respectively). Although synchro is primarily recognized for long underwater episodes that activate complex adjustment mechanisms for respiratory compensation (i.e., apnea) (Naranjo et al., 2006), thrusts (also known as body-jumps) are among the most well-recognized elements of synchro. Body-jumps are characteristic elements of the sport in which the athlete uses synchro-swimming techniques to rise as high as possible from the water (i.e., to jump out of the water). Two of the most important and most widely used synchro thrusts are the Barracuda and the Boost. The athlete performing the Barracuda must reach the highest possible vertical position throughout the leg-first jump from the water (Figure 1). Throughout the Boost, the subject quickly rises headfirst from the water and lifts the arms to the final position before the upward momentum of the jump is lost (Figure 2). Due to the clear need for the rapid and maximal production of force, both moves are considered power moves and are therefore related to explosive strength.

Explosive strength (muscular power) is recognized as a highly important motor ability in numerous sports (Buchheit et al., 2010b; Hakkinen, 1993; Mirkov et al., 2004; Paruzel-Dyja et al., 2007). In brief, it is the ability to generate a maximal level of strength in the shortest period of time. The most widely recognized sport-related manifestations of explosive strength are throwing (Granados et al., 2007) and jumping (Sattler et al., 2011). As one of the most important physical capacities in sport, vertical jumping performance is assessed with a variety of tools ranging from sophisticated electronic measuring instruments (e.g., force platforms, contact mats, or photocells) to popular field-testing procedures (e.g., the Sargent jump test or the Abalakov test). In such assessments, different types of jumps may be performed (e.g., a squat jump, countermovement jump (CMJ), or repeated jumps), and different parameters may be measured (e.g., maximum jump height, maximum reach height, relative jump height, flight time, mechanical power, or body displacement) (Sattler et al., 2011). Many studies have examined the results of standard jumping procedures, such as the CMJ and squat jump, in relation to reliability, validity and applicability (Buchheit et al., 2010a; Cordova and Armstrong, 1996; Glatthorn et al., 2011; Markovic et al., 2004; Slinde et al., 2008). However, recent studies have noted the need for insight into sport-specific jumping procedures (Sattler et al., 2011). In short, those studies showed that jumping performance should be tested more precisely, and thus more applicably, in sport-specific jumps (in their case, the spike-jump and block-jump in volleyball), than in standard jumps (i.e., the squat jump and CMJ).

The synchro thrusts briefly introduced previously (the Barracuda and Boost) are clearly comparable to jumps made in groundsports such as volleyball and basketball, as both types of movement rely on the same type of muscular activation (i.e., the generation of maximal strength in the shortest time possible) and, consequently, on the same physiological origin (i.e., muscle fiber types) (Komi and Ishikawa 2009). Although the Barracuda and Boost are generally recognized as the synchronized swimming equivalent of vertical jumps in groundsports, we have found no investigations that evaluated the reliability and validity of these two sport-specific testing procedures. Furthermore, to the best of our knowledge, the relationship of synchro thrusts to competitive achievement in synchro has not been studied previously. Although we recognize that synchro consists of different characteristic elements that merit study, we have focused this investigation on only one group of sport-specific elements – body jumps.

The aim of the present study was to determine the reliability and validity of two synchro-specific jumping tests, the Barracuda and Boost, compared with the frequently used and systematically validated countermovement-jump test (CMJ). In addition, the results were correlated with anthropometric variables and competitive achievement to establish their applicability and predictive validity in synchronized swimming.

## Materials& Methods

### Participants

The subjects were 22 female synchro swimmers (age 16 to 18 years; body height 167.32±4.1 cm; body mass 56.32±3.21 kg) who had been training in synchro for 4 to 8 years.

### Measures

The sampled variables were anthropometric measures, the results of jumping performance, and overall competitive achievement in the solo routine at the most recent national-level competition.

The anthropometric variables included body height (BH), body mass (BM), and the body composition variables of lean body mass (LBM), percent LBM (LBM%), body fat mass (BF), and percent BF (BF%). BH was measured with a scale fixed to a wall, and BM was measured with a digital scale. Body composition was measured using the MALTRON BF 900 analyzer (Maltron International Ltd, Rayleigh, UK). In short, Maltron works on bio-electrical impedance at 50kHz by measuring the impedance (resistance plus reactance - at this frequency, the current passes across cell membranes) of the body to a safe alternating current. The Maltron Analyzer uses the tetrapolar method, in which four electrodes are applied to the right side of the body on the hand, wrist, foot and ankle (Tomljanovic et al. 2011)

Each subject was tested throughout one session. All participants were tested within a week, two to three weeks before the competition at which competitive achievement was recorded (see previous text).

The jumping performance evaluations included CMJ and two synchro-specific jumping tests, the Barracuda and the Boost. The subject, wearing standard tennis shoes, performs a CMJ on the ground. The CMJ begins with the subject standing in an upright position with the arms resting on the hips. A rapid downward movement to approximately 90° knee flexion is immediately followed by a rapid upward, vertical movement, whose aim is to move the body as high off the ground as possible. The CMJ occurs as one continuous movement. The test was measured using Optojump testing equipment (Microgate, Bolzano, Italy), and three trials were conducted. In this study, the CMJ is chosen as a test of standard (ground) jumping ability because the subjects are familiar with the test procedure (i.e., a very similar testing procedure is used as a test of explosive strength throughout the national physical education curriculum).

The Barracuda and Boost were measured in a 2.20-meter deep swimming pool. The Barracuda starts from a back pike position, head down, with the legs perpendicular to the surface of the water (Figure 1a). When performing the Barracuda, the athlete executes a rapid, vertical upward movement of the legs and hips as the body unrolls to obtain a maximal vertical position (Figure 1b). The Boost starts from a vertical swimming position (Figure 2a). The subject rapidly rises headfirst from the water and lifts the arms to the final position before upward momentum is lost (Figure 2b).To test both elements, the subjects were instructed to perform a rapid vertical rise from the water with the aim of raising as much of the body (legs) as possible above the surface of the water. For each trial, we recorded the height of the toes (for the Barracuda) and fingers (for the Boost) relative to the water surface. The height was measured with a measuring scale fixed to the false-start rope in the first lane of the swimming pool. Because of the water density, the athlete stops briefly in the final position of the test. This position allowed the examiners to determine the height effectively. Each examinee performed three trials for the Boost and for the Barracuda, and each trial was measured by a different examiner (all experienced synchro swimming coaches). This procedure allowed us to define the reliability for sport-specific jumps.

After the reliability analysis, the best result from each subject was kept for further analysis. In addition, the results of the Boost and Barracuda were normalized for BH.

### Statistical analysis

Descriptive statistical parameters (means, standard deviations) were calculated for all the variables for each individual trial and for the overall results (the subject-specific best result) of all jumping tests. Average inter-trial correlation coefficients (IIR) and Cronbach’s alpha reliability coefficients (CA) were used to determine the between-subject reliability of the jumping tests. The within-subject variation for each of the tests was determined by calculating the coefficient of variation (CV). An analysis of variance (ANOVA) for repeated measures was used to detect any systematic bias between individual trials for each jumping test. To determine the factorial validity of the jumping tests, the inter correlation matrix of the three tests was factorized with a principal-components factor analysis. The number of significant components was determined with the Kaiser-Guttman criterion. The correlations between jumping tests with the significant factor component were used to determine the factorial validity of the tests. The final results of the anthropometric measures and the jumping achievements were correlated with competitive achievement by calculating the Pearson correlation coefficients. All of the coefficients were considered significant at 95% (p < 0.05). Statsoft’s Statistica (ver. 7.0) was used for all calculations.

## Results

The results of the reliability analysis showed high reliability for both sport-specific tests and for CMJ. However, the reliability parameters showed higher between-subject reliability and somewhat lower within-subject reliability for the sport-specific tests than for the standard ground jump (CMJ) (Table 1). ANOVA detected no systematic changes between trials (no significant differences between trials for each of the tests).

Factor analysis revealed one significant factor, with no evident difference in factor structure if the results for the Boost and Barracuda were expressed as absolute achieved results or if the results were normalized for BH. All three tests were significantly correlated with the significant component; therefore, the Barracuda and Boost meet all the prerequisites for being defined as measures of specific explosive strength in synchronized swimming (Table 2).

Correlation analysis showed small to moderate significant correlations between anthropometric measures and achievement and between body composition and achievement in the Barracuda and Boost. Only LBM was significantly correlated with the results of CMJ (Table 3).

**Figure 3:**
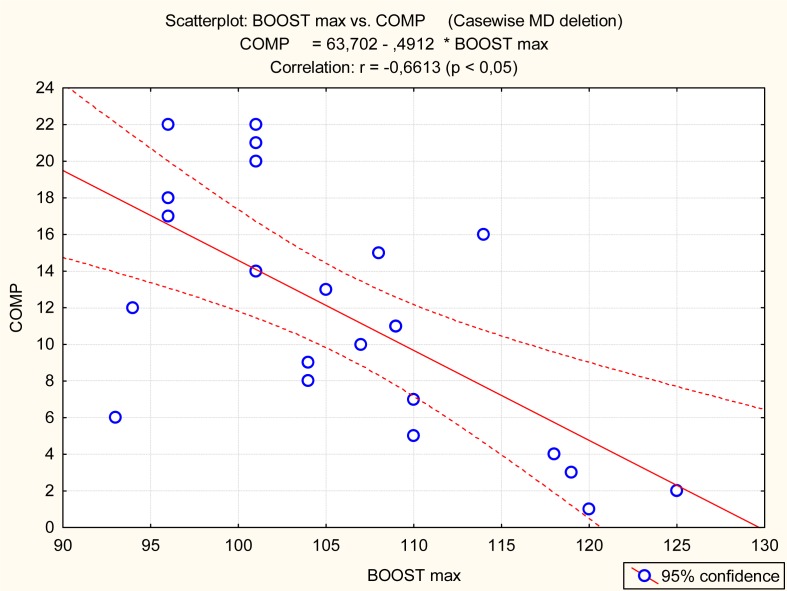
Graphical presentation of the relationship between Boost (BOOST max) and competitive achievement (COMP)

**Figure 4:**
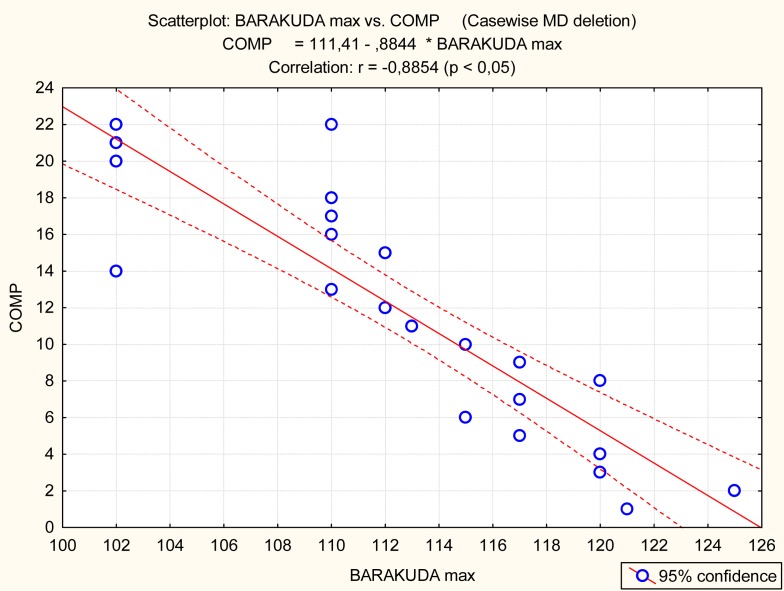
Graphical presentation of the relationship between Barracuda (BARAKUDA max) and competitive achievement (COMP)

**Figure 5:**
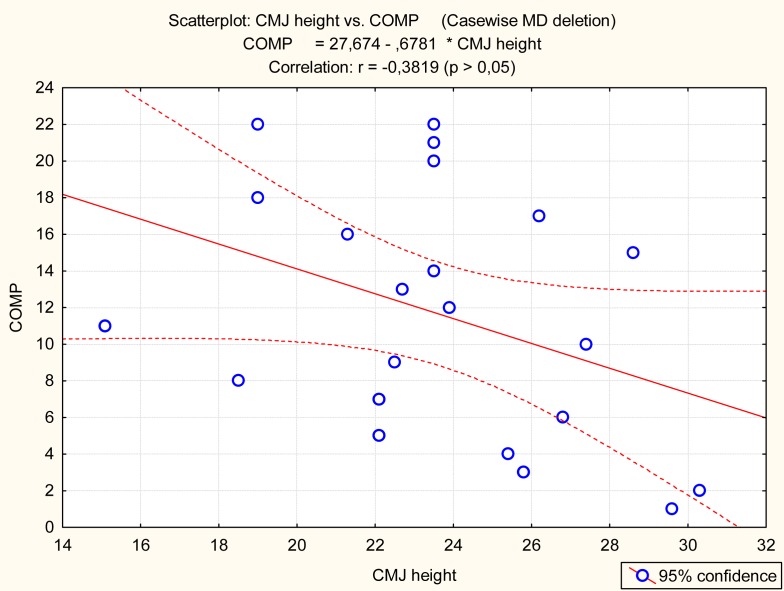
Graphical presentation of the relationship between CMJ (CMJ height) and competitive achievement (COMP)

The Barracuda and Boost, but not CMJ, were significantly related to the competitive achievement of synchro swimmers (Table 3 and Figures 3–5).

## Discussion

Different sports rely on different motor and functional abilities that play important roles in the overall achievement of athletes (Cipryan and Gajda, 2011; Strzala et al., 2007). Consequently, athletes and coaches pay special attention to the testing and development of characteristic fitness segments that allow an athlete to compete at an advanced level. One of the main prerequisites for useful testing is to have accurate and reliable measuring tools. Studies have regularly investigated the reliability and validity of different measuring tools in sports (Duncan et al., 2005; Sattler et al., 2011). Generally, higher reliability parameters were found if (a) the investigators sampled subjects who were familiar with the testing procedures (Duncan et al., 2005) and (b) the athletes were tested on sport-specific tests (Sattler et al., 2011). Therefore, although the Barracuda and Boost had not previously been studied for their reliability, their relatively high reliability was not surprising. First, the Barracuda and Boost are synchro sport-specific elements. Therefore, even if the subjects had not performed those elements as part of a testing procedure prior to this study, they had performed them frequently during training and competition. Consequently, it is probable that the swimmers we tested were more familiar with the sport-specific jumps than with CMJ. Therefore, the high between-subject reliability of the sport-specific jumps is understandable. The somewhat lower within-subject reliability of the sport-specific tests (note that sport-specific jumps have a somewhat higher CV than the CMJ) is most likely unrelated to different evaluations by the examiners (because ANOVA found no systematic differences between judges or trials). The probable explanation of this outcome is that sport-specific elements performed in water depend on certain “uncontrollable” factors, such as water instability (i.e., waves) and/or the position of the arms/legs at the highest point of the jump (see Figures). However, we consider that the way in which we have expressed the final score on each of the tests (i.e., the best of three trials) diminished the influence of within-subject variation on the final test result.

In our study, the factorial validity of the sport-specific tests Barracuda and Boost was established with the same statistical procedure that was previously used to study the factorial validity of standard (Markovic et al., 2004) and sport-specific jumping tests (Sattler et al., 2011). Two sport-specific jumps and CMJ (standard jump), were included in the factor analysis. Due to the equal physiological basis of all three jumps (i.e., rapid excitation of the maximum possible number of motor units), we hypothesized that CMJ, a jump performed on the ground, would correlate with Barracuda and Boost, jumps that are performed in the water. Factor analysis extracted only one significant factor, with all three tests significantly projected, allowing us to define an appropriate factor validity for the sport-specific tests. The results show that the sport-specific tests should be considered measures of sport-specific explosive strength. Additionally, we normalized the results for the Barracuda and Boost by dividing the absolute achievement on each of the tests by the subject’s BH. This adjustment allowed us to calculate an additional factor analysis, but it did not change the results. In both factor analyses, however, the results showed a lower projection of the CMJ on the main component. From the mathematical basis and logic of the factor analysis, it is clear that the cross-correlations between the synchro-specific jumps (Barracuda and Boost) and CMJ are not as high as the inter-correlations between the Barracuda and the Boost. One can argue that if all tests possess the same physiological basis, as previously suggested, then there is no clear reason for such a discrepancy in the correlations between the variables. The reason for the inconsistency in the relationships can be explained with the following argument. In jumps performed on the ground, such as the CMJ, the athlete tends to utilize a mechanical impulse. Impulse is defined as the change in momentum when an athlete’s body collides with the earth at take-off. The imperceptible change in the momentum of the earth is set against the much greater change generated by the jumper’s body, resulting in body displacement (e.g., a jump). Body mass and, to a greater extent, body composition are factors that influence the utilization of the mechanical impulse to achieve as high a jump as possible (Sekulic et al., 2005). Therefore, the positive influence of LBM (as a generator of force) and the negative influence of BF indices (as a ballast mass) on the CMJ results are expected. However, our results revealed some unexpected relationships between body composition measures and synchro power moves. We found that swimmers with higher BF are more successful in performing the Boost (note the significant positive correlation between these two variables). These somewhat-unexpected results are not difficult to explain if the characteristics of synchro sport are considered. In synchro, the BF should not always be observed as ballast mass because body fat is less dense than water. Therefore, a higher proportion of BF assures that a larger proportion of the body is above the surface of the water at the beginning of the Boost thrust (see Figure 2a). This positioning decreases the resistance of the water during the first phase of the jump (i.e., a smaller portion of the body is submerged), allowing the athlete to perform the jump rapidly and to reach (i.e., jump) higher. As clear supporting evidence, we note the negative correlation between the LBM% and Boost performance. Briefly, previous studies already noted that LBM% is related to body density (Cordain and Kopriva, 1991), which, in turn, is directly related to lower buoyancy and, in our case, to poorer Boost performance. However, note that the BF percentage of the sampled synchronized swimmers was relatively low and that an average BF of 13.09% (see Results) places them among the leanest female athletes (Gonzalez-Rave et al., 2011; Hasan et al., 2007; Malousaris et al., 2008; Neumayr et al., 2003). The relationship between body composition and achievement in Barracuda is similar, with the difference that body fat in this jump does not influence the volume of the body that is submerged at the beginning of the test (see Figure 2a) but instead increases buoyancy during the performance of the thrust (Zamparo et al., 1996). Consequently, the negative influence of the LBM is not as pronounced as in the Barracuda.

Previous studies rarely related specific variables to competitive achievement in synchro swimming. Aside from a recent study (Gabrilo et al., 2011) which showed a significant relationship between pulmonary function and competitive achievement in synchro swimming, we have found only one study that examines the relationships between physiological characteristics (including isokinetic muscle strength, muscle endurance, and flexibility) and competitive achievement in synchro (Yamamura et al., 1999). In that study, the authors found physiological characteristics to be important determinants of the performance score. However, to our knowledge, our investigation is the first to relate sport-specific motor tests to competitive achievement in synchronized swimming. We find this approach to be important for two reasons. First, sport-specific fitness tests are more convenient and applicable than standard (i.e., general) fitness tests because athletes are highly familiar with the movement patterns of sport-specific tests (Sattler et al., 2011), a principle that is directly supported by the results of our study (see the previous discussion on reliability). Second, athletes are regularly interested in exercise testing that is directly related to their performance (Laure, 1997). Their interest results in their increased engagement and commitment during testing and therefore to more relevant results. As we have shown, the two sport-specific tests included in this study are significantly related to competitive achievement, whereas CMJ is not. This result did not surprise us because the Barracuda and the Boost are strongly emphasized in the judging of solo synchro routines. More precisely, choreography (e.g., lifts, throws) that are a regular part of a team routine can partially diminish the importance of Barracuda and Boost performance if swimmers perform duets and/or as a team. During a solo performance, however, the athlete’s competitive achievement depends directly on the quality, accuracy and height of the jumps.

## Conclusions

The Barracuda and Boost are considered power moves in synchronized swimming; previously, however, their reliability and validity in sport-specific testing had not been determined. The results of this study show that both moves can be used reliably as a diagnostic tool in the analysis of sport-specific explosive strength. However, it must be emphasized that both elements are highly technical and require advanced sport-specific technique; therefore, reproducibility is accepted only among athletes who are experienced in synchro training and competition (i.e., at least 3–4 years of systematic training). The results of both tests were related to competitive achievement. Therefore, progress in each of the validated tests could be related to improvement in performance. We advise that synchronized swimming coaches use the suggested testing procedures described herein to define the sport-specific power capacities of athletes. The present study demonstrated high applicability of the two sport-specific tests, and we suggest that further investigations of sport-specific tests in synchro swimming be conducted. Due to the highly specific but also potentially dangerous apnea episodes in synchro, it would be particularly important to study specific tests of diving performance.

## Figures and Tables

**Table 1:** Descriptive statistics (Mean; SD – standard deviation) for all measured variables; reliability analysis for jumping tests (CA – Cronbach Alpha; IIR – inter-trial correlation; CV – coefficient of variation)

	**Mean**	**SD**	**CA**	**IIR**	**CV**
Barracuda_trial1_(cm)	110.27	7.29			
Barracuda_trial2_(cm)	110.32	6.19			
Barracuda_trial3_(cm)	110.55	7.18			
Barracuda_max_(cm)	112.82	6.69	0.92	0.84	0.09
Boost_trial1_(cm)	102.95	8.68			
Boost_trial2_(cm)	102.05	10.45			
Boost_trial3_(cm)	101.36	11.72			
Boost_max_(cm)	106.00	9.00	0.95	0.89	0.08
CMJ_trial1_(cm)	24.41	3.66			
CMJ_trial2_(cm)	23.55	3.55			
CMJ_trial3_(cm)	23.61	4.01			
CMJ_max_(cm)	23.65	3.73	0.89	0.79	0.05
BH (cm)	167.32	4.10			
BM (kg)	56.32	3.21			
BF% (%)	13.09	1.55			
BF (kg)	7.44	1.54			
LBM% (%)	87.21	2.00			
LBM (kg)	49.01	5.23			

Barracuda – Barracuda synchronized swimming thrust; Boost – Boost synchronized swimming jump; CMJ – countermovement jump; BH – body height; BM – body mass; BF - body fat; LBM - lean body mass

**Table 2:** Factor analysis of the applied jumping tests (Factor) for the absolute achievements (max) and normalized for body height (norm)

	**Factor**		**Factor**
Boost_max_	0.87	Boost_norm_	0.86
Barracuda_max_	0.84	Barracuda_norm_	0.79
CMJ_max_	0.71	CMJ_max_	0.66
Expl.Var	1.97	Expl.Var	1.81
Prp.Totl	0.81	Prp.Totl	0.77

Barracuda – Barracuda synchronized swimming thrust; Boost – Boost synchronized swimming jump; CMJ – countermovement jump; Expl.Var – variance of the significant factor; Prp.Totl – total proportion of the explained variance

**Table 3:** Pearson correlation of the anthropometric (body composition) variables and competitive achievement with jumping performance (maximal results on three trials)

	Boost	Barracuda	CMJ
BH	0.48^*^	0.39	0.23
BW	0.49^*^	0.37	0.12
BF%	0.54^*^	0.51^*^	0.19
BF	0.60^*^	0.49^*^	0.16
LBM%	−0.63^*^	−0.42	0.33
LBM	0.44^*^	0.33	0.46^*^
COMP^¥^	−0.66^*^	−0.89^*^	−0.38

Barracuda – Barracuda synchronized swimming thrust; Boost – Boost synchronized swimming jump; CMJ – countermovement jump; BH – body height; BM – body mass; BF - body fat; LBM - lean body mass; COMP – competitive achievement;

* denote significant coefficients at p < 0.05;

¥ competitive achievement is oppositely scaled (lower numerical value presents better competitive result - placement)

## References

[b1-jhk-32-135] Bante S, Bogdanis GC, Chairopoulou C, Maridaki M (2007). Cardiorespiratory and metabolic responses to a simulated synchronized swimming routine in senior (>18 years) and comen (13–15 years) national level athletes. J Sports Med Phys Fitness.

[b2-jhk-32-135] Buchheit M, Spencer M, Ahmaidi S (2010). Reliability, usefulness, and validity of a repeated sprint and jump ability test. Int J Sports Physiol Perform.

[b3-jhk-32-135] Buchheit M, Mendez-Villanueva A, Delhomel G, Brughelli M, Ahmaidi S (2010). Improving repeated sprint ability in young elite soccer players: repeated shuttle sprints vs. explosive strength training. J Strength Cond Res.

[b4-jhk-32-135] Cipryan L, Gajda V (2011). The Influence of Aerobic Power on Repeated Anaerobic Exercise in Junior Soccer Players. Journal of Human Kinetics.

[b5-jhk-32-135] Cordain L, Kopriva R (1991). Wetsuits, body density and swimming performance. Br J Sports Med.

[b6-jhk-32-135] Cordova ML, Armstrong CW (1996). Reliability of ground reaction forces during a vertical jump: implications for functional strength assessment. J Athl Train.

[b7-jhk-32-135] Duncan MJ, Al-Nakeeb Y, Nevill AM (2005). Influence of familiarization on a backward, overhead medicine ball explosive power test. Res Sports Med.

[b8-jhk-32-135] Gabrilo G, Peric M, Stipic M (2011). Pulmonary function in pubertal synchronized swimmers: 1-year follow-up results and its relation to competitive achievement. Med Probl Perform Art.

[b9-jhk-32-135] Glatthorn JF, Gouge S, Nussbaumer S, Stauffacher S, Impellizzeri FM, Maffiuletti NA (2011). Validity and reliability of Optojump photoelectric cells for estimating vertical jump height. J Strength Cond Res.

[b10-jhk-32-135] Gonzalez-Rave JM, Arija A, Clemente-Suarez V (2011). Seasonal changes in jump performance and body composition in women volleyball players. J Strength Cond Res.

[b11-jhk-32-135] Granados C, Izquierdo M, Ibanez J, Bonnabau H, Gorostiaga EM (2007). Differences in physical fitness and throwing velocity among elite and amateur female handball players. Int J Sports Med.

[b12-jhk-32-135] Hakkinen K (1993). Changes in physical fitness profile in female basketball players during the competitive season including explosive type strength training. J Sports Med Phys Fitness.

[b13-jhk-32-135] Hasan AA, Reilly T, Cable NT, Ramadan J (2007). Anthropometric profiles of elite Asian female handball players. J Sports Med Phys Fitness.

[b14-jhk-32-135] Komi PV, Ishikawa M, Maughan RJ Physiology and Biocehmistry. The Olympic Text of Science in Sport.

[b15-jhk-32-135] Laure P (1997). How do elite athletes perceive exercise testing?. Science & Sports.

[b16-jhk-32-135] Malousaris GG, Bergeles NK, Barzouka KG, Bayios IA, Nassis GP, Koskolou MD (2008). Somatotype, size and body composition of competitive female volleyball players. J Sci Med Sport.

[b17-jhk-32-135] Markovic G, Dizdar D, Jukic I, Cardinale M (2004). Reliability and factorial validity of squat and countermovement jump tests. J Strength Cond Res.

[b18-jhk-32-135] Mirkov DM, Nedeljkovic A, Milanovic S, Jaric S (2004). Muscle strength testing: evaluation of tests of explosive force production. Eur J ApplPhysiol.

[b19-jhk-32-135] Naranjo J, Centeno RA, Carranza MD, Cayetano M (2006). A test for evaluation of exercise with apneic episodes in synchronized swimming. Int J Sports Med.

[b20-jhk-32-135] Neumayr G, Hoertnagl H, Pfister R, Koller A, Eibl G, Raas E (2003). Physical and physiological factors associated with success in professional alpine skiing. Int J Sports Med.

[b21-jhk-32-135] Paruzel-Dyja M, Iskra J, Zajac A (2007). Somatic and fitness endowment of sprinting stride in the context of developmental changes and diverse sport activities. Journal of Human Kinetics.

[b22-jhk-32-135] Sattler T, Sekulic D, Hadzic V, Uljevic O, Dervisevic E (2011). Vertical jumping tests in volleyball: reliability, validity and playing-position specifics. J Strength Cond Res.

[b23-jhk-32-135] Sekulic D, Zenic N, Markovic G (2005). Non linear relationships between anthropometric and motor-endurance variables. CollAntropol.

[b24-jhk-32-135] Slinde F, Suber C, Suber L, Edwen CE, Svantesson U (2008). Test-retest reliability of three different countermovement jumping tests. J Strength Cond Res.

[b25-jhk-32-135] Strzala M, Tyka A, Krezalek P (2007). Physical endurance and swimming technique in 400 metre front crawl race. Journal of Human Kinetics.

[b26-jhk-32-135] Tomljanovic M, Spasic M, Gabrilo G, Uljevic O, Foretic N (2011). Effects of five weeks of functional vs. traditional resistance training on anthropometric and motor performance variables. Kinesiology.

[b27-jhk-32-135] Yamamura C, Zushi S, Takata K, Ishiko T, Matsui N, Kitagawa K (1999). Physiological characteristics of well-trained synchronized swimmers in relation to performance scores. Int J Sports Med.

[b28-jhk-32-135] Zamparo P, Antonutto G, Capelli C, Francescato MP, Girardis M, Sangoi R, Soule RG, Pendergast DR (1996). Effects of body size, body density, gender and growth on underwater torque. Scand J Med Sci Sports.

[b29-jhk-32-135] Zenic N, Peric M, Zubcevic NG, Ostojic Z, Ostojic L (2010). Comparative analysis of substance use in ballet, dance sport, and synchronized swimming: results of a longitudinal study. Med Probl Perform Art.

